# Intercellular Communication by Vascular Endothelial Cell-Derived Extracellular Vesicles and Their MicroRNAs in Respiratory Diseases

**DOI:** 10.3389/fmolb.2020.619697

**Published:** 2021-01-29

**Authors:** Shota Fujimoto, Yu Fujita, Tsukasa Kadota, Jun Araya, Kazuyoshi Kuwano

**Affiliations:** ^1^ Division of Respiratory Disease, Department of Internal Medicine, The Jikei University School of Medicine, Tokyo, Japan; ^2^ Department of Translational Research for Exosomes, The Jikei University School of Medicine, Tokyo, Japan

**Keywords:** vascular endothelial cell, extracellular vesicle, exosome, microvesicle, microRNA, respiratory disease

## Abstract

Respiratory diseases and their comorbidities, such as cardiovascular disease and muscle atrophy, have been increasing in the world. Extracellular vesicles (EVs), which include exosomes and microvesicles, are released from almost all cell types and play crucial roles in intercellular communication, both in the regulation of homeostasis and the pathogenesis of various diseases. Exosomes are of endosomal origin and range in size from 50 to 150 nm in diameter, while microvesicles are generated by the direct outward budding of the plasma membrane in size ranges of 100–2,000 nm in diameter. EVs can contain various proteins, metabolites, and nucleic acids, such as mRNA, non-coding RNA species, and DNA fragments. In addition, these nucleic acids in EVs can be functional in recipient cells through EV cargo. The endothelium is a distributed organ of considerable biological importance, and disrupted endothelial function is involved in the pathogenesis of respiratory diseases such as chronic obstructive pulmonary disease, pulmonary hypertension, and acute respiratory distress syndrome. Endothelial cell-derived EVs (EC-EVs) play crucial roles in both physiological and pathological conditions by traveling to distant sites through systemic circulation. This review summarizes the pathological roles of vascular microRNAs contained in EC-EVs in respiratory diseases, mainly focusing on chronic obstructive pulmonary disease, pulmonary hypertension, and acute respiratory distress syndrome. Furthermore, this review discusses the potential clinical usefulness of EC-EVs as therapeutic agents in respiratory diseases.

## Introduction

Respiratory diseases are leading causes of death and disability in the world (Soriano et al., 2020). Noxious agents such as cigarette smoke and air pollution are the causes and aggravating factors of a variety of respiratory diseases (Renzoni et al., 2014; Vij et al., 2018). They impose an immense worldwide health burden, with hundreds of millions of people suffering from respiratory diseases each year (Soriano et al., 2020). Therefore, there is an urgent need to elucidate the pathogenesis of respiratory diseases and to develop novel therapies to treat them. To date, a number of studies have demonstrated the involvement of the vascular endothelium in the pathogenesis of respiratory diseases (Dai et al., 2018; Huertas et al., 2018; Leligdowicz et al., 2018). Pulmonary vascular endothelium is strategically located to separate blood from air and to filter the blood before it enters systemic circulation. Then, endothelial cells (ECs) play essential roles in maintaining lung homeostasis by optimizing gas exchange, reducing vascular tone, coordinating of blood flow control, and modulating hypoxic vasoconstriction (Cahill and Redmond, 2016; Hsia et al., 2016). Moreover, extensive researches have confirmed that vascular endothelium is a highly specialized metabolically active organ possessing numerous physiological, immunological, and synthetic functions (Colbert and Schmidt, 2016; Wong et al., 2017). Among the various important functions of vascular endothelium is the secretion of extracellular vesicles (EVs), which are key players in intercellular communication (Fujita et al., 2016). EVs, which include exosomes and microvesicles, are released from almost all cell types. EVs contain various compositions of nucleic acids, namely mRNAs, microRNAs (miRNAs), and DNA fragments, in addition to proteins and lipids (Kubo, 2018). The most common proteins that compose EVs are tetraspanins (CD9, CD63, CD81, and CD82), which are a protein superfamily that interacts with many protein partners including major histocompatibility complex (MHC) molecules and integrins. Tetraspanins are involved in the organization of functional molecular complexes called tetraspanin-enriched microdomains (Willms et al., 2018). EVs also contain heat shock proteins (Hsp70 and Hsp90) that associate with antigen presentation (Zhang et al., 2019b). Membrane-associated proteins (annexins, GTPases, and flotillin), multivesicular bodies (MVBs) synthesis proteins (ALG-2-interacting protein X (Alix), tumor susceptibility gene 101 (Tsg101)), and major MHC classes I and II are also present in EVs from most cell types (Hessvik and Llorente, 2018). Furthermore, the lipid composition of EVs reveals sphingomyelin, cholesterol, ceramide, and glycophospholipids (Skotland et al., 2019). EV compositions have the ability to play important roles in homeostasis and pathological processes, such as angiogenesis, inflammation, and senescence (Fujita et al., 2015b; Andrews and Rizzo, 2016; Takasugi, 2018). Notably, EVs derived from ECs (EC-EVs) have attracted significant attention as biological mediators and potential therapeutic targets in respiratory diseases (Kadota et al., 2016; Lanyu and Feilong, 2019). However, the precise relevance is incompletely understood. In this review, we summarize the pathological roles of vascular miRNAs contained in EC-EVs in respiratory diseases, mainly focusing on chronic obstructive pulmonary disease (COPD), pulmonary hypertention (PH), and acute respiratory distress syndrome (ARDS). Moreover, we also discuss the potential clinical usefulness of EVs as therapeutic agents in respiratory diseases.

## Extracellular Vesicles

In recent decades, EVs have emerged as essential players of intercellular communication. Membrane-bound vesicles outside cells were first identified in mice 50 years ago (Anderson, 1969). Pan B. T. and Johnstone R. M. reported that small vesicles are released from sheep reticulocytes in 1983 and they proposed to define such small vesicles as exosomes in 1987 (Pan and Johnstone, 1983; Johnstone et al., 1987). In 2007 and 2010, exosomes were reported to contain both mRNAs and miRNAs, which can be delivered to other cells and can function in their new location (Valadi et al., 2007; Kosaka et al., 2010). Following these studies, numerous additional EV studies have been conducted in various fields, including lung disease pathogenesis (Fujita et al., 2015a; Genschmer et al., 2019; Kadota et al., 2020). EVs are released into the extracellular environment by almost all cell types, including epithelial cells, fibroblasts, ECs, cancer cells, stem cells, and immune cells (Whiteside, 2018; Li et al., 2019; Mohan et al., 2020). Moreover, EVs have been isolated from diverse bodily fluids, including blood, urine (Street et al., 2017), saliva (Machida et al., 2015), breast milk (Qin et al., 2016), bronchoalveolar lavage fluid (Admyre et al., 2003), semen (Madison et al., 2015), amniotic fluid (Dixon et al., 2018), ascites (Yun et al., 2019), and sputum (Njock et al., 2019). Therefore, EVs reflect various pathological conditions, and have attracted attention as a biomarker for lung diseases (Kadota et al., 2017).

The two major categories of EVs include exosomes and microvesicles (also referred to as ectosomes and microparticles), which are classified on the basis of their biogenesis and secretion mechanisms (Raposo and Stoorvogel, 2013). Exosomes are of endosomal origin and have a size range of 50-150 nm in diameter. Exosomes are generated through a process that involves double invagination of the plasma membrane and the formation of intracellular MVBs containing intraluminal vesicles, which are released into the extracellular space by the fusion of the peripheral membrane of the MVBs with the plasma membrane (Kalluri and LeBleu, 2020). Exosomes can deliver their components to target cells through clathrin- or caveolin-dependent endocytosis, phagocytosis, micropinocytosis, lipid raft-mediated internalization, and membrane fusion (Mulcahy et al., 2014). This results in the delivery of proteins and RNAs into the membrane or cytosol of the recipient cell, and the transfer of this material can regulate gene expression and fate decisions of target cells (Lakkaraju and Rodriguez-Boulan, 2008). By contrast, microvesicles are vesicles generated by the direct outward budding of the plasma membrane with a size range of 100-2000 nm in diameter. Microvesicles are rich in phosphatidylserine, and their basic cargo includes membrane components similar to those of the parent cell membrane (Hargett and Bauer, 2013). Although the origins of exosomes and microvesicles have been defined, current technologies cannot reliably separate EVs (Merchant et al., 2017). The International Society for Extracellular Vesicles (ISEV) advocates “extracellular vesicle” (EV) as the generic term for particles released from the cell that are all lipid bilayer-enclosed extracellular structures (Thery et al., 2018). In this review, we use the term EVs.

The significance of EVs lies in their capacity to deliver their cargoes to various sites in the body and influence a wide variety of biological processes of the recipient cells (Fujita et al., 2016). Additionally, the compositions and functions of EVs are modulated in response to environmental stressors. Specifically, in the lung, it has been reported that hypoxia, infection, and cigarette smoke increase the numbers of EVs and alter their compositions (de Jong et al., 2012; Hargett and Bauer, 2013; Fujita et al., 2015a). Therefore, EVs are important paracrine mediators not only in homeostasis but also in disease pathogenesis. These properties suggest that EVs can be useful as biomarkers for the diagnosis, prognosis, and therapeutic response of various lung diseases.

## Non-coding RNAs in EVs

A non-coding RNA (ncRNA) is a generic term for RNA that does not code for proteins (Fatima and Nawaz, 2017). NcRNAs can influence numerous molecular targets to drive specific cell biological responses and fates (Cech and Steitz, 2014; Anastasiadou et al., 2018b). In 1993, lin4, a first ncRNA that negatively regulates the level of LIN-14 protein, was discovered in a nematode (Lee et al., 1993). In 1998, RNA interference (RNAi), a sequence-specific suppression phenomenon induced by double-stranded RNA, was discovered (Hannon, 2002). In 2000, it was revealed that another ncRNA, let-7, is widely conserved in many species, including humans, which dramatically improves the recognition of short ncRNAs (Reinhart et al., 2000). To date, the development of next-generation sequencing and RNA sequencing has led to the discovery of a multitude of ncRNA species, such as small interfering RNAs (siRNAs), P-element-induced wimpy testes (piwi)-interacting RNAs (piRNAs), enhancer RNAs (eRNAs), miRNAs, and the long-noncoding RNAs (lncRNAs) (Cech and Steitz, 2014; Espinosa, 2016; Anastasiadou et al., 2018b). They have crucial roles in the regulation of the genome, from transcription into mRNA, to mRNA processing, to translation into proteins (Vencken et al., 2015). As a result, ncRNAs are involved in various processes, including cell proliferation, tissue differentiation, metabolic regulation, the cell cycle, apoptosis, and metastasis (Guo et al., 2014). Thus, dysregulation of ncRNAs may lead to dysfunctions and diseases (Anastasiadou et al., 2018a). It has been reported that EVs contain various types of functional ncRNAs (Xie et al., 2019). Here, we mainly focus on EV miRNAs that have been widely explored and have potential for use as biological mediators and therapeutic targets for various diseases.

MiRNAs are endogenously expressed, single-stranded RNAs that are approximately 18-25 nucleotides in length (Mohr and Mott, 2015). As mentioned above, miRNAs are important regulators in several biological processes. Indeed, the development and progression of various diseases have been associated with miRNA expressions (Rupani et al., 2013). The biogenesis of miRNAs takes place through a multi-step process, and miRNAs regulate gene expression via binding to the 3'-untranslated region of mRNAs to induce degradation and translational repression, thereby suppressing the expression of target genes (Bartel, 2018; Becker et al., 2019). One miRNA may target many mRNAs, and conversely, one mRNA may be regulated by several miRNAs (Matsuyama and Suzuki, 2019).

It has been reported that miRNAs can stably exist in bodily fluids (Salido-Guadarrama et al., 2014). Extracellular miRNAs are protected from decomposition and stabilized by being packed into EVs, loaded into high density lipoproteins, and bound to argonaute 2 protein, which is the key effector protein of miRNA-mediated silencing (Ha and Kim, 2014; Tabet et al., 2014). EV miRNA and mRNA profiles differ from those of the parental cells, suggesting that cells have an active selection mechanism for EV cargo (Zhang et al., 2017). The miRNAs are not randomly incorporated into EVs, because the proportion of miRNAs is higher in EVs than in parental cells, and EV miRNA expression levels are altered under different physiological conditions (Goldie et al., 2014). These results indicate that parental cells have a sorting mechanism that guides specific intracellular miRNAs to enter EVs (Zhang et al., 2015). Sorted miRNAs are delivered by EVs to recipient cells, regulating biological processes such as cell differentiation, proliferation, apoptosis, and metabolism (Kim et al., 2017). Therefore, EV miRNAs are associated with inflammatory conditions, metabolic disorders, malignancies, and autoimmune disorders (Adyshev et al., 2014). Although EVs have various functional compositions except miRNAs, EV miRNA cargo has the potential to regulate gene expression both locally and remotely, and are therefore drawing attention in various fields (Chen et al., 2017; O'Brien et al., 2018; Cavallari et al., 2019).

## Endothelial Cell-Derived EVs in Endothelial Physiology

The vascular endothelium plays a wide variety of critical roles in the maintenance of vascular homeostasis (Pi et al., 2018). Vessels deliver metabolites and oxygen to tissues and export waste products to sustain the well-being of an organism (Helms et al., 2018). The endothelium is the monolayer of ECs lining the lumen of blood or lymph vessels, which are able to differentiate into arterial, venous, and lymphatic cells during development and act as a barrier between the blood or lymph and tissues (Hulshoff et al., 2019). The endothelium is also a distributed organ of considerable biological potential that not only serves as a barrier, but also perform other distinctive biologic functions (Rohlenova et al., 2018). In addition, the ECs interact with circulating cells and cells present in the vascular wall. Interestingly, ECs display functional heterogeneity in different tissues and organs, which suggests that ECs alter their function for adapting microenvironment (Kruger-Genge et al., 2019). In the lungs, ECs have a variety of physiological functions, including optimizing gas exchange, the reduction of vascular tone, the coordination of blood flow, and the modulation of hypoxic vasoconstriction (Suresh and Shimoda, 2016). ECs can shed EVs directly into the bloodstream and deliver them to distant sites (Hromada et al., 2017). Thus, the functions of ECs are partially performed through delivering EVs.

Several unique proteome and nucleic acid cargoes have been found in EC-EVs (Letsiou and Bauer, 2018). EC-EVs are defined according to the expression of endothelial membrane-specific antigens. CD62E (E-selectin) and CD144 (vascular endothelial (VE)-cadherin) are specific markers for EC-EVs (Dignat-George and Boulanger, 2011). Angiotensin converting enzyme (ACE) can also serve to identify specific EC-EVs subpopulations, as it is abundantly expressed in pulmonary capillary endothelium (Takei et al., 2019b). There are other common EC-EV markers, such as CD54 (ICAM-1: intercellular adhesion molecule 1), CD62P (P-selectin), CD31 (PECAM: platelet-endothelium cell adhesion molecule), CD105 (endoglin, a growth-related protein), and CD146 (MCAM: melanoma cell adhesion molecule) (Choi et al., 2015). Since these are also expressed in platelet-derived microparticles, EC-EV specificity is guaranteed by the absence of the platelet-specific glycoprotein Ib marker CD42b (Chironi et al., 2009).

**FIGURE 1:**
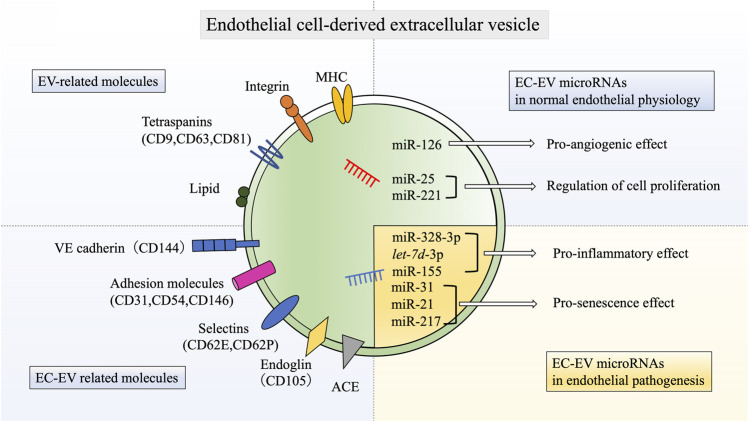
Schematic molecular components of endothelial cell-derived extracellular vesicles. EC-EVs have various EV-related molecules, such as tetraspanins, MHC, integrins, and lipids. EC-EVs also have unique endothelial markers, including VE-cadherin, E-selectin, P-selectin, PECAM, ICAM-1, MCAM, endoglin, and ACE. In addition, EC-EVs also contain various miRNAs that have biological functions in both normal endothelial physiology and pathogenesis. EC: endothelial cell, EV: extracellular vesicle, miR: microRNA, CD144: vascular endothelial (VE)-cadherin, CD31: platelet-endothelium cell adhesion molecule (PECAM), CD54: intercellular adhesion molecule 1(ICAM-1), CD146: melanoma cell adhesion molecule (MCAM), CD62E: E-selectin, CD62P: P-selectin, ACE: angiotensin converting enzyme, MHC: major histocompatibility complex.

Regarding the nucleic acid cargo of EC-EVs, an analysis of miRNAs in both EC-EVs and parental cells has demonstrated that different miRNAs are distributed differentially between cells and EVs (van Balkom et al., 2015). In this study, the 10 most abundant miRNAs in EVs are miR-10b, *let-7i,* miR-126, miR-100, miR-27b, miR-30a, miR-25, miR-221, miR-191, and miR-411. Among these, angiogenesis-related miRNAs, such as miR-126, miR-25, and miR-221, are higher in abundance in EVs than in cells. Data indicate that these miRNAs may be sorted within EVs on purpose. Mechanistically, miR-126-3p regulates angiogenesis via modulating angiogenic growth factors, such as vascular endothelial growth factor (VEGF) and fibroblast growth factor (FGF)-2, and promotes blood vessel formation by repressing the expression of sprouty-related protein-1 (Spred-1), an intracellular inhibitor of angiogenic signaling (Wang et al., 2008; Todorova et al., 2017). Furthermore, miR-25 and miR-221 affect EC proliferation (Nicoli et al., 2012; Mujahid et al., 2013; Zhang et al., 2019a). These miRNA cargos permit EC-EVs to play pivotal roles in regulating angiogenesis, possibly resulting in homeostatic control during endothelial physiology (Figure 1).

## Endothelial Cell-Derived EVs in Endothelial Pathogenesis

Endothelial dysfunction is a key initiating event in several pathological conditions. Vascular disorders increase permeability that can lead to vascular leakage and edema formation, perturb the balance between vasoconstriction and vasodilation, initiate the expression of adhesion molecules for inflammatory cell recruitment, and activate pro-inflammatory and pro-coagulant molecules (Rajendran et al., 2013; Fernandez-Hernando and Suarez, 2018; Huertas et al., 2018). Pulmonary ECs are exposed to various stresses, such as physiologic mechanical stress, endotoxins, and cigarette smoke. These stress stimuli impair endothelial functions and induce EV secretion (Heiss et al., 2008; Gordon et al., 2011; Liu et al., 2014; Letsiou et al., 2015). Furthermore, it has been reported that elevated levels of CD42b^-^/CD31^+^ endothelial microparticles are associated with vascular disease and endothelial dysfunction in patients with coronary artery disease, renal failure, and metabolic disorders (Amabile et al., 2005; Chironi et al., 2009; Wu et al., 2020). These evidences indicate that EC-EVs are also involved in endothelial dysfunction and various types of disease pathogenesis. Therefore, we will describe the pathological roles of EC-EVs in terms of endothelial dysfunction, especially inflammation, and senescence (Table 1).

**TABLE 1:** Key properties of EC-EVs in the endothelial pathogenesis.

	EC-EV stimulation	Recipient cell	EC-EV composition	EV function	References
Inflammation	TNF-α	hAE	Not specified	ICAM-1 induction	(Curtis et al., 2009)
	TNF-α	HPMEC	Not specified	NF-κβ pathway activation	(Liu et al., 2017)
	TNF-α, IL-1β, IFN-γ, and LPS	b.End5	miR-328-3p and *let-7d*-3p	VEGF-B induction and pro-inflammatory effect	(Yamamoto et al., 2015)
	ox-LDL	HUVEC	miR-155	Monocyte activation	(He et al., 2018)
Senescence	Cell senescence	mAEC	Not specified	Senescence induction in a ROS-dependent manner	(Burger et al., 2012)
	Cell senescence	HUVEC	miR-31	Osteogenic differentiation inhibition	(Weilner et al., 2016)
	Cell senescence	HUVEC	miR-21-5p and miR-217	Senescence induction and cell replicative rate reduction	(Mensa et al., 2020)

EV: extracellular vesicle, EC: endothelial cell, miR: microRNA, TNF-α: tumor necrosis factor-α, IL: interleukin, IFN: interferon, LPS: lipopolysaccharide, ox-LDL: oxidized low-density lipoprotein, ICAM-1: intercellular adhesion molecule-1, NF-κβ: nuclear factor-kappa β, ROS: reactive oxygen species, VEGF: vascular endothelial growth factor, hAEC: human aortic endothelial cell, mAEC: mouse aortic endothelial cell, b.End5: brain-derived endothelial cell line, HUVEC: Human umbilical vein endothelial cell, HPMEC: human pulmonary microvascular endothelial cell.

### Inflammation

The number of EVs is elevated in acute and chronic inflammatory diseases, such as sepsis, atherosclerosis, and diabetes mellitus (Helmke and von Vietinghoff, 2016; Freeman et al., 2018; Murao et al., 2020). In addition, emerging evidence implicates EVs as a causal and contributing factor in altering vascular cell phenotypes by mediating proinflammatory signaling pathways (Andrews and Rizzo, 2016). In human aortic ECs, EVs secreted by tumor necrosis factor-α (TNF-α) stimulation can act as paracrine mediators, inducing an inflammatory response (Curtis et al., 2009). Similarly, TNF-α-derived EVs activate the nuclear factor kappa-light-chain-enhancer of activated B cells (NF-κβ) pathway in human pulmonary microvascular ECs, thereby facilitating the inflammatory response (Liu et al., 2017). When exposed to inflammatory stimuli, ECs can sort many inflammation-related miRNAs, especially miR-328-3p and *let-7d-3p*, into EC-EVs, resulting in pathological neovascularization and vascular leakage (Yamamoto et al., 2015). Moreover, the atherosclerosis inducer, oxidized low-density lipoprotein (ox-LDL), is able to upregulate miR-155 expression in ECs. This miRNA is enriched in ox-LDL-induced EC-EVs, and miR-155-containing EVs regulate inflammation in association with atherosclerosis by monocyte activation (He et al., 2018). Thus, EC-EVs have the potential to induce inflammation through the composition of their cargoes.

### Senescence

Senescence is a cellular response that induces irreversible growth arrest accompanied by distinct phenotypic alterations, such as chromatin rearrangement, metabolic reprogramming, autophagy modulation, and the implementation of a complex proinflammatory secretome, collectively referred as the senescence-associated secretory phenotype (SASP) (Campisi and d'Adda di Fagagna, 2007). Senescence has important roles in normal development, in maintaining tissue homeostasis, and in limiting tumor progression. However, senescence has also been implicated as a major cause of age-related diseases (van Deursen, 2014). Senescent ECs have been implicated in the pathogenesis of endothelial dysfunction. Several lines of evidence suggest that senescent ECs are involved in responses to different types of damage, such as telomere erosion, ROS-mediated DNA damage, vascular inflammation, mitochondrial dysfunction, and the renin-angiotensin-aldosterone system, which are characteristic of dysregulation of vascular tone, increases in endothelium permeability, and impairment of angiogenesis and mitochondrial biogenesis, leading to endothelial dysfunction.

Importantly, accumulating evidence suggests that EVs derived from senescent cells can regulate the phenotype of recipient cells similar to SASP (Kadota et al., 2018). Our recent study showed that human primary lung fibroblasts from patients with idiopathic pulmonary fibrosis (IPF) have a senescent phenotype and secrete increased levels of EVs containing miR-23b-3p and miR-494-3p, which has been shown to induce mitochondrial damage, increase epithelial-cell DNA damage responses, and further lead to senescence (Kadota et al., 2020). This indicates that senescent fibroblasts control their surroundings by reinforcing senescence in a paracrine manner through EV cargoes like SASP. EC-EVs have been found to be an important mediator associated with cell senescence in the microenvironment (Takasugi, 2018). It has been reported that EVs derived from senescent ECs induce premature senescence in normal ECs in a ROS-dependent manner (Burger et al., 2012). Indeed, senescent EC-derived EVs have been reported to contain miR-31, miR-21, and miR-217, which act as pro-senescence effectors to ECs (Weilner et al., 2016; Alique et al., 2017; Lee et al., 2019; Mensa et al., 2020). In the endothelium, we can speculate that EVs derived from senescent ECs may transfer pro-senescence signals, resulting in disease progression and causing comorbidities through spreading cellular senescence.

**FIGURE 2:**
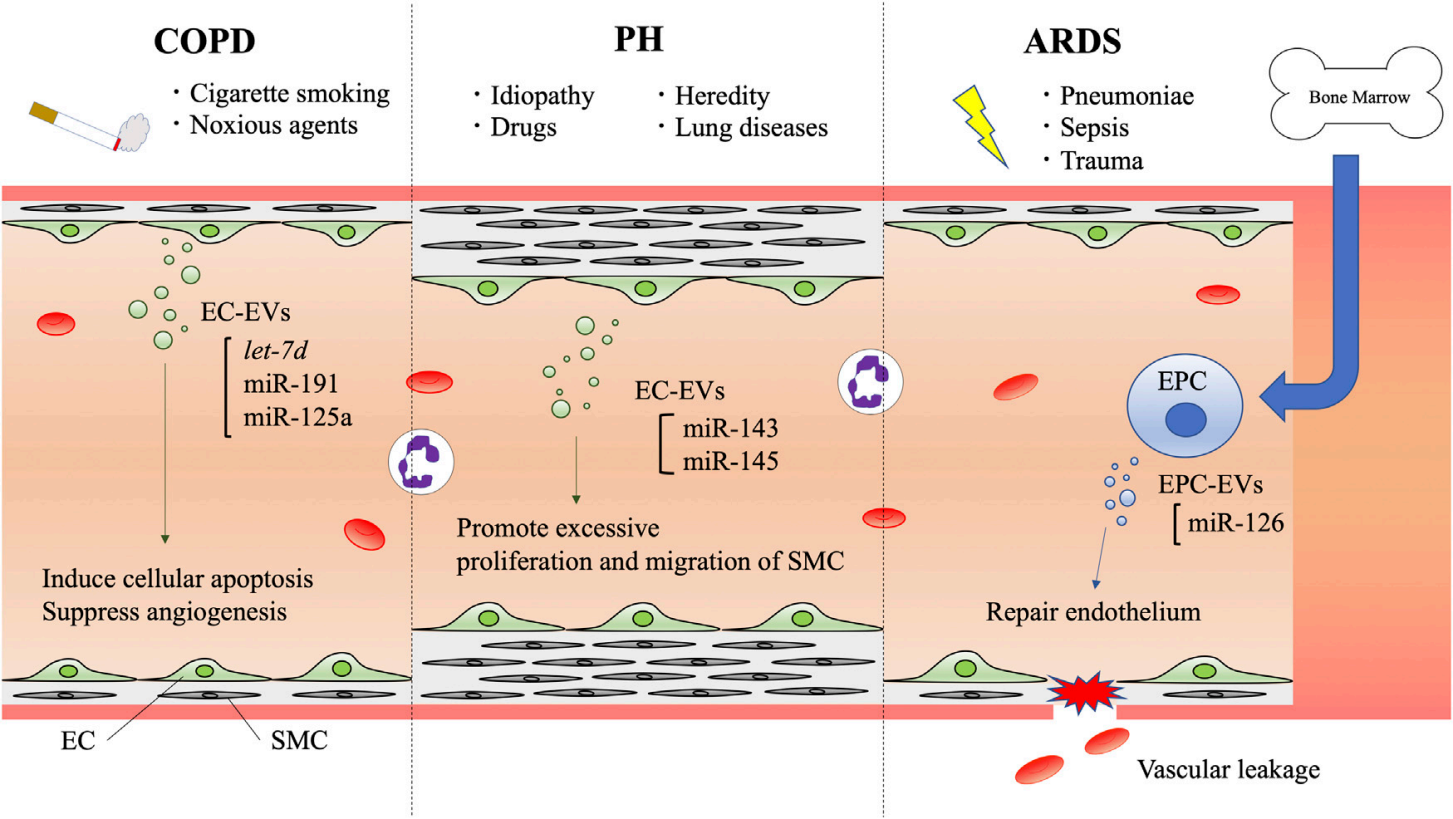
Schematic diagram of endothelial cell-derived extracellular vesicle communication in respiratory diseases. EVs are secreted to the bloodstream by vascular endothelial cells or endothelial progenitor cells. In COPD, cigarette smoke and noxious agents induce EV secretion, and their cargoes are involved in inducing cellular apoptosis and suppressing angiogenesis. In PH, several factors such as heredity, drugs, and other lung diseases are known to cause PH. Histological features have revealed EC proliferation and SMCs with vascular remodeling. ECs and SMCs crosstalk with each other via EVs. EC-EV miR-143 and miR-145 have the potential to promote excessive proliferation and migration of SMCs. In ARDS, pneumoniae, sepsis, and trauma can induce vascular leakage. EPCs derived from bone marrow secrete EVs that contain miR-126, which can help to repair endothelium. COPD: chronic obstructive pulmonary disease, PH: pulmonary hypertension, ARDS: acute respiratory distress syndrome, EC: endothelial cell, EPC: endothelial progenitor cell, SMC: smooth muscle cell, EV: extracellular vesicle, miR: microRNA.

There are many additional reports describing various functions of EC-EVs in association with homeostasis and pathogenesis, and EV miRNAs seem to play a major role in these functions. Next, we will review the functions of EC-EVs and their miRNAs in respiratory disease pathogenesis (Figure 2).

## Endothelial Cell-Derived EVs in Chronic Obstructive Pulmonary Disease

COPD is a common disease and a leading cause of morbidity and mortality worldwide. COPD is characterized by chronic inflammation and airflow limitation that is not fully reversible. The common symptoms are dyspnea, cough, and sputum production (Vogelmeier et al., 2017). COPD is usually caused by inhaled cigarette smoke and other noxious particles or gases. In addition, host factors are also relevant, for instance genetic abnormalities, those born with low lung function, and abnormal lung development (Stocks and Sonnappa, 2013). Most patients with COPD in the general population are probably affected by both multiple genetic factors and environmental factors. However, approximately 1–5% of COPD cases have α-1-antitrypsin deficiency which causes emphysema in nonsmokers (Takei et al., 2019a). Although the pathogenesis of COPD is very complicated, including chronic inflammation, proteinase/anti-proteinase imbalance, oxidative stress, and alveolar cell apoptosis, there are several reports showing that the vascular endothelium is deeply involved in the pathogenesis of COPD (Fischer et al., 2015; Schuliga, 2015; Lomas, 2016). For instance, cigarette smoking initiates pulmonary vascular malfunction through direct injury to ECs (Polverino et al., 2017), resulting in a persistent inflammatory response and pulmonary emphysema in lung tissues (Chen et al., 2012). Indeed, the presence of endothelial dysfunction has been confirmed in smokers and in patients with COPD (Piccari et al., 2020).

EC-EVs are associated with pulmonary and systemic vascular damage or other biological activities relevant to COPD pathogenesis. Endothelial damage has been reported to occur during acute exacerbations of COPD, and EVs can affect this process (Takahashi et al., 2012). COPD exacerbations are crucial events in the course of the disease that accelerate the progressive decline in lung function and increase the risk of death (Beasley et al., 2012; Song et al., 2014). The clusters of differentiation CD144^+^, CD31^+^, and CD62E^+^ EV reflect endothelial damage during COPD exacerbations, indicating that EV levels in patients with COPD exacerbation are significantly higher than in stable patients (Takahashi et al., 2012). Although further research on the role of these EVs is needed to elucidate their pathophysiology, it is likely that EC-EVs are involved in the mechanisms of COPD development.

There are various reports on the association between smoking and EVs in the pathogenesis of COPD (Hobbs and Hersh, 2014; Kara et al., 2016; Strulovici-Barel et al., 2016). Cigarette smoke extract (CSE) has been confirmed to reduce the level of α1-antitrypsin secretion by ECs, suggesting that CSE may also inhibit α1-antitrypsin transport via EC-EVs (Lockett et al., 2014). Our group showed that EVs from CSE-induced bronchial epithelial cells can cause airway fibrosis in COPD pathogenesis through autophagy suppression by EV miR-210 (Fujita et al., 2015a). It is not surprising that EC-EVs function and containing miRNAs can be altered in response to cigarette smoke exposure. Indeed, circulating EC-EVs released during cigarette smoke exposure are significantly enriched in vascular disease-related miRNAs, such as *let-7d*, miR-191, and miR-125a. EC-EVs containing these miRNAs may play key roles in inflammation and vascular remodeling changes in response to cigarette smoke (Serban et al., 2016). *Let-7d* induces cellular apoptosis and suppresses the proliferation, migration, and tubulogenesis of ECs (Ji et al., 2019). MiR-125a and miR-191 also promote cell apoptosis and suppress angiogenesis (Svensson et al., 2014; Gu et al., 2017; Du et al., 2019). These evidences suggest that EC-EVs and their miRNAs are involved in COPD pathogenesis and disease progression.

EVs released from the vascular endothelium circulate throughout the body and influences various distant sites. Therefore, EC-EVs not only participate in COPD pathogenesis, but are expected to be involved in the development of its various comorbidities. Systemic manifestations and comorbidities of COPD include skeletal muscle wasting, cardiovascular disease, osteoporosis, anemia, metabolic syndrome, and depression (Breyer et al., 2014; Caram et al., 2016; Inoue et al., 2016; Martinez Rivera et al., 2016; To et al., 2017; Xiong et al., 2018). As an example, it has been reported that EVs may play a role in linking COPD to cardiovascular comorbidities. Increased EC-EV levels in patients with COPD are related to prevalence of cardiovascular disease, even when compensating for confounding factors (Garcia-Lucio et al., 2018). Although further studies will be needed to elucidate the pathogenic EC-EV functions in linking COPD to the comorbidities, circulating EC-EVs in patients with COPD might exacerbate systemic endothelial injury, potentially leading to the development of cardiovascular disease. As another example, several miRNAs, such as miR-21, miR-134a, miR-133, and miR-206, have been proposed as potential biomarkers of sarcopenia in patients with COPD, and the levels of miR-21 and miR-206 in particular are strongly correlated with hand-grip strength (Qaisar et al., 2020). This suggests that EC-EVs may be involved in COPD-related sarcopenia pathogenesis, since miR-21 is enriched in EVs derived from senescent ECs as mentioned previously. Future research will elucidate the pathogenesis of COPD and the mechanism of its complications through EC-EV cargo. It is expected that the EC-EVs and their miRNAs will be used as biomarkers and targets of treatment.

## Endothelial Cell-Derived EVs in Pulmonary Hypertension

PH is a severe disease characterized by elevated pulmonary arterial pressure and pulmonary vascular resistance, resulting in right ventricular hypertrophy, progressive fibrosis, right ventricular failure, and low cardiac output, leading to increase morbidity and mortality (Wei et al., 2013). Clinically, PH is divided into five groups, which are pulmonary arterial hypertension (PAH), pulmonary hypertension due to left heart disease, pulmonary hypertension due to lung diseases, chronic thromboembolic pulmonary hypertension, and pulmonary hypertension due to other disorders (Simonneau et al., 2019). Although several drugs are available for the treatment of PH, there is no cure for this disease, and current therapies are limited in reversing vascular remodeling. Thus, the prognosis remains poor, particularly for patients with severe PH (Hoeper et al., 2017). The histological features reveal proliferation of ECs and smooth muscle cells (SMCs) with irreversible vascular remodeling. EC dysfunction is considered to be a critical initiating factor in the development and progression of PAH. Furthermore, it is known that hypoxia and inflammation contribute to the development of endothelial damage (Rabinovitch et al., 2014). Then, sustained vasoconstriction and vascular remodeling result in increased pulmonary arterial pressure and pulmonary vascular resistance (Ranchoux et al., 2018).

There is accumulating evidence for a critical role of EC-EVs and their miRNAs in the pathogenesis of PH (Wei et al., 2013; de la Cuesta et al., 2019; Khandagale et al., 2020). Circulation levels of EC-EVs are increased in patients with PH, and levels correlate with pulmonary arterial pressure, pulmonary vascular resistance, thrombotic tendency, and mortality (Bakouboula et al., 2008; Aliotta et al., 2013). CD31^+^ and CD144^+^ EVs correlate with mean pulmonary arterial pressure and brain natriuretic peptide (BNP), suggesting that these EC-EVs are likely indicators of the hemodynamic severity of PH. CD62E^+^ EVs do not correlate with hemodynamic severity, but they do with C-reactive protein. This suggests that subpopulations of EC-EVs may reflect the diversity of injuries that occur in PH (Amabile et al., 2008).

Additionally, several miRNAs are involved in the pathogenesis of PH. MiR-143 and miR-145 are enriched in EVs secreted by Kruppel-like factor 2-transduced ECs, and are transferred from ECs to SMCs (Hergenreider et al., 2012; Doddaballapur et al., 2015). These miRNAs, which are increased in patients with PH and in a hypoxic mouse model of PH, promote excessive proliferation and migration of pulmonary artery SMCs, leading to PH development (Caruso et al., 2012; Mohsenin, 2016). Inhibition of miR-143 and miR-145 protects against the development of PH, suggesting that there is an important role for miR-143 and miR-145 in PH pathogenesis (Deng et al., 2015). As shown in these reports, EC-EVs and their miRNAs are involved in the pathology of PH.

## Endothelial Cell-Derived EVs in Acute Respiratory Distress Syndrome

ARDS is a devastating and rapidly progressive respiratory disorder that is characterized by increased release of inflammatory mediators and disruption of the integrity of alveolar and vascular endothelial barriers (Thompson et al., 2017). The pathogenesis of ARDS is the destruction of the epithelial and endothelial layers caused by a variety of pulmonary or systemic inflammatory stimuli, including bacteria, viruses, thrombin, mechanical stretching, cytokines, and chemokines (Williams et al., 2017; Batah and Fabro, 2020; Xie et al., 2020). Cytokines and inflammatory mediators play essential roles in initiating and mediating ARDS, with inflammatory responses including endothelial adhesion molecule expression, the release of pro-inflammatory mediators, EC cytoskeleton rearrangement, and apoptosis (Maniatis et al., 2008; Wang et al., 2019). These stimuli contribute to lung injury by promoting EC barrier disruption and excessive translocation of inflammatory immune cells into the lungs (Curtis et al., 2013). In addition, ECs are activated by inflammatory stimuli, such as interleukin (IL)-1, IL-6, TNF-α, and oxidative stress in ARDS. Activated ECs promote microvascular injury by releasing complement and proinflammatory cytokines. Then, this injury disrupts EC tight junctions and adherens junctions, resulting in increased alveolar capillary permeability (Lu et al., 2012; Guo et al., 2016). Therefore, endothelial dysfunction leads to increasing vascular permeability and subsequent pulmonary edema and respiratory failure (Bellani et al., 2016; Fan et al., 2018).

Recent studies have demonstrated that EC-EVs are involved in EC dysfunction and the pathogenesis of ARDS (Li et al., 2015a; Shaver et al., 2017). It has been reported that EC-EVs induce endothelial dysfunction by causing the impairment of endothelium vasodilation. In an animal acute lung injury (ALI) model, plasminogen activator inhibitor-1 (PAI-1)-induced EC-EVs lead to the compromise of the alveolar-capillary barrier, inducing pulmonary edema and stimulating interstitial neutrophil accumulation (Densmore et al., 2006). In addition, when EC-EVs are injected intravenously into mice, it triggers pulmonary and systemic release of IL-1β and TNF-α and enhances lung injury in a lipopolysaccharide (LPS)-induced ALI (Buesing et al., 2011). Other studies have shown that LPS increases the number of circulating EC-EVs that express CD144, CD62E, CD54, or CD31 compared to healthy controls in a murine model (Li et al., 2015b; Yu et al., 2017). Indeed, EC-EVs are elevated in the plasma of patients with ARDS compared to healthy controls and in patients with cardiogenic pulmonary edema. These facts suggest that EC-EVs can contribute to the development of ARDS (Chen et al., 2017).

Patients with ARDS may develop acute renal failure, heart failure, thrombosis, and eventually multiple organ failure (Evans and Zhao, 2017; Araujo et al., 2019; Fowler et al., 2019). Intriguingly, Coronavirus Disease 2019 (COVID-19) infection, which can lead to ARDS and thrombosis, may be related to EC-EVs (Mehta et al., 2020; Zaid et al., 2020). It has been suggested that COVID-19 virus infects ECs by binding the ACE-2 receptor and using it for internalization, then the virus directly attacks microvascular ECs (Zhang et al., 2020). In addition, there is evidence that EC-EVs transfer ACE-2 to recipient cells (Wang et al., 2020). These facts suggest that EC-EVs may contribute to spread of this virus and support internalization (Hassanpour et al., 2020). Thus, understanding the function of EC-EVs in COVID-19-related ARDS is conducive to early identification and proper treatment of ARDS.

## Endothelial Progenitor Cell-Derived EV Therapy

Currently, endothelial progenitor cell (EPC)-derived EVs (EPC-EVs) are used as therapeutics for various experimental diseases, including ARDS. Asahara *et al.* reported that EPCs can be isolated from human peripheral blood and bone marrow by utilizing markers for hematopoietic stem cells. It has thus been confirmed that these cells can differentiate into ECs (Asahara et al., 1997; Peters, 2018). Circulating EPCs derived from bone marrow migrate toward vascular lesions and contribute to vasculogenesis (Asahara et al., 1999). EPCs probably participate in maintaining vascular homeostasis and facilitate vascular repair (Kalka et al., 2000). Moreover, EPCs have been shown to exert their beneficial effects through paracrine mechanisms, including through the transmission of mediators via EVs. EPC-EVs could be incorporated into ECs to enhance their proliferation, migration, and angiogenic tubule formation (Zhang et al., 2016). Moreover, these EVs have beneficial effects on various preclinical models, such as sepsis, acute kidney injury, and ischemia (Cantaluppi et al., 2012; Ranghino et al., 2012; Zhou et al., 2018).

To date, there are no approved pharmacological treatments for ARDS. Therefore, EVs and their miRNAs have attracted attention as a pathological mediator and treatment method for ARDS in recent years (Fujita et al., 2018). In preclinical studies, intratracheal administration of EPC-EVs has also been shown to reduce lung injury through decreased local inflammatory cytokines, lung permeability, and neutrophil migration (Zhou et al., 2019). Previous evidences have indicated that miR-126, which is an endothelial-specific miRNA and is highly expressed in ECs, is involved in the regulation of vascular integrity and angiogenesis (Guo et al., 2016; Wu et al., 2016). Remarkably, miR-126-3p and -5p are abundant in EPC-EVs and have the ability to reduce the expression of genes associated with the development of ARDS (Zhou et al., 2019). In fact, recent studies have shown that miR-126-3p maintains the integrity of the endothelial barrier by targeting phosphoinositide-3-kinase regulatory subunit 2 (PIK3R2) and Spred-1, leading to the stabilization of VE-cadherin. Additionally, miR-126-5p inhibits inflammatory alarmin high mobility group box-1 (HMGB1) and the permeability factor VEGFα, attenuating endothelium barrier disruption and decreasing vascular permeability (Xi et al., 2017; Zhou et al., 2019). Conversely, suppression of miR-126 has been shown to increase vascular permeability (Pepini et al., 2010; Fernandez-Hernando and Suarez, 2018). These studies indicate that EPC-EVs may have novel therapeutic potential for ARDS through unique miRNA-related mechanisms.

## Conclusion

The vascular endothelium is a distributed organ of considerable biological importance and has been shown to contribute to vascular homeostasis and disease. As shown in this review, EC-EVs are associated with vascular homeostasis and pathological conditions such as inflammation and senescence, and also play an important role in the pathogenesis of respiratory diseases such as COPD, PH, and ARDS. In normal endothelial physiology, EC-EVs function by maintaining homeostasis, and it has been found that the EVs are mainly involved in angiogenesis. Angiogenesis is facilitated by the miRNAs contained in EC-EVs, which regulate angiogenic signals and vascular EC proliferation, resulting in homeostatic control of vasculature physiology. In endothelial pathogenesis, EC-EVs alter their secretion and components in response to various stimuli, and they further regulate inflammation and senescence phenotypes, resulting in the progression of respiratory diseases and their comorbidities. In terms of EV therapeutics, EPC-EVs have the potential to reduce lung damage through local reduction of inflammatory cytokines, lung permeability, and neutrophil migration in ARDS pathogenesis.

**FIGURE 3:**
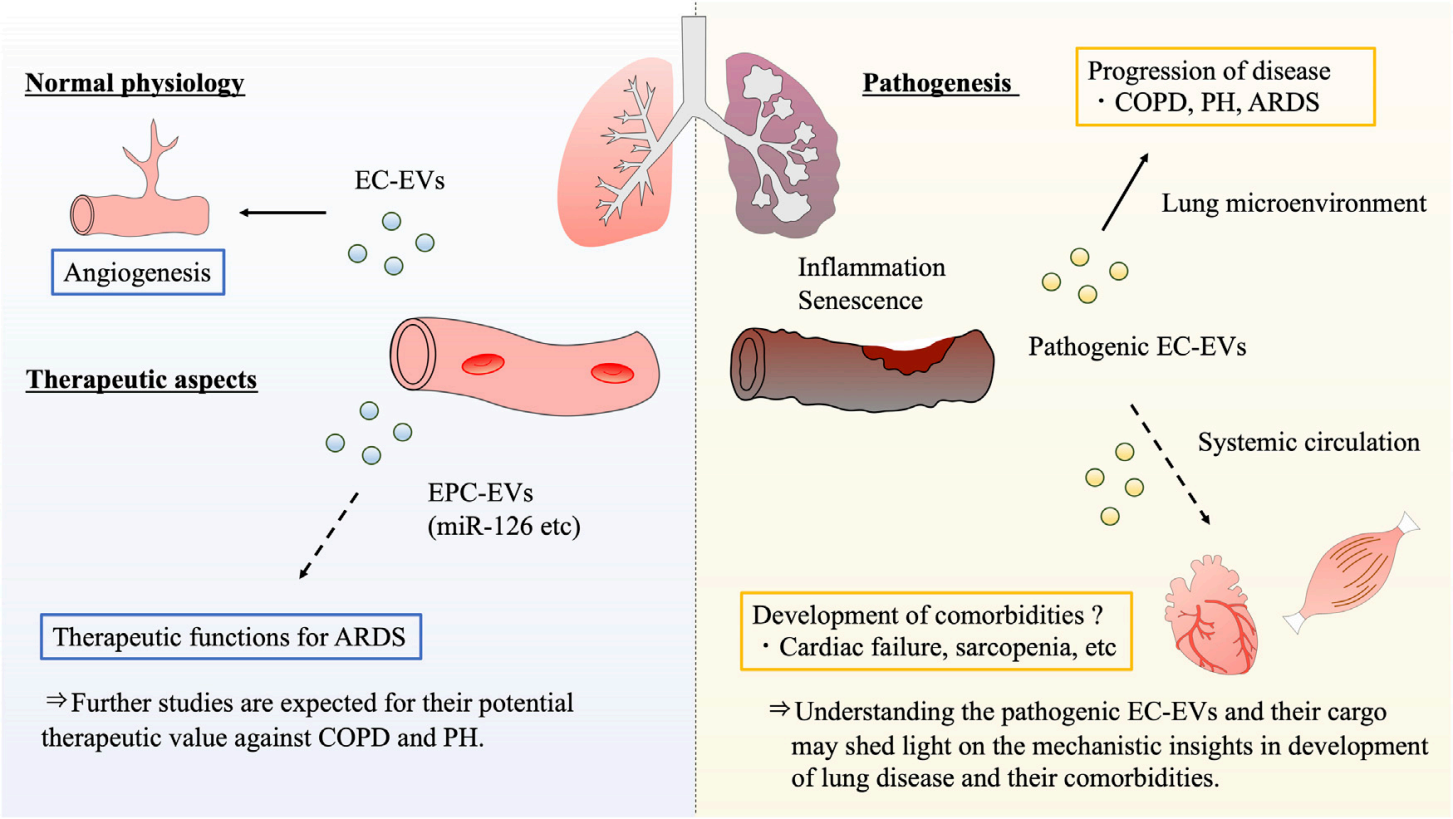
Summary and future perspectives of endothelial cell-derived extracellular vesicle potential. Based on the previous evidences, it is clear that EC-EVs have the functions of regulating angiogenesis in normal endothelial physiology. With regard to therapeutic aspect, EPC-EVs may have the potential to be developed as novel treatments for various respiratory diseases. Recent studies indicate that EPC-EVs have therapeutic functions for ARDS through unique miRNA-related action mechanisms. On the other hand, pathogenic EC-EVs further promote inflammation and senescence phenotypes in the endothelial microenvironment, resulting in the progression of respiratory diseases such as COPD, PH, and ARDS. Notably, EC-EVs circulate throughout the body, the EVs exert influences over various sites. As future perspectives, pathogenic EC-EVs can form the respiratory disease pathogenesis, and are expected to be involved in the development of their comorbidities. COPD: chronic obstructive pulmonary disease, PH: pulmonary hypertension, ARDS: acute respiratory distress syndrome, EC: endothelial cell, EPC: endothelial progenitor cell, EV: extracellular vesicle.

EC-EVs are useful for mechanistic research and biomarker discovery, because blood vessels are located in places that are easier to approach than other organs. It is now thought that EC-EVs are involved in not only respiratory disease progression, but also in the development of systemic comorbidities through circulating cargoes. Thus, EVs and their miRNAs may be clinically useful, but the mechanism of their effects has not yet been fully elucidated. Understanding the pathological effects of EC-EVs and their cargo may bring mechanistic insights to the development of lung disease comorbidities, such as cardiovascular disease and muscle atrophy. Furthermore, the identification of pathological EC-EVs will lead to the discovery of novel progressive or predictive biomarkers for lung diseases and their systemic complications. Since the vascular endothelium is present in almost all organs, it has been researched in various fields and has the potential to be applied in different fields that support each other. In conclusion, we speculate that research on vascular endothelium and EC-EVs of lung diseases and their comorbidities will be useful not only for understanding pathophysiology, but also for developing novel treatments for various diseases (Figure 3).
